# Erythropoietin treatment alleviates ultrastructural myelin changes induced by murine cerebral malaria

**DOI:** 10.1186/1475-2875-11-216

**Published:** 2012-06-28

**Authors:** Casper Hempel, Poul Hyttel, Trine Staalsø, Jens R Nyengaard, Jørgen AL Kurtzhals

**Affiliations:** 1 Centre for Medical Parasitology at Department of International Health, Immunology and Microbiology, University of Copenhagen and Department of Clinical Microbiology, Copenhagen University Hospital, Denmark; 2 Department of Veterinary Clinical and Animal Sciences, Faculty of Health and Medical Sciences, University of Copenhagen, Copenhagen, Denmark; 3 Department of Veterinary Clinical and Animal Sciences Faculty of Health and Medical Sciences, University of Copenhagen, Denmark

**Keywords:** Cerebral malaria, *Plasmodium berghei* ANKA, Demyelination, Neurological sequelae, Erythropoietin

## Abstract

**Background:**

Cerebral malaria (CM) is a severe complication of malaria with considerable mortality. In addition to acute encephalopathy, survivors frequently suffer from neurological sequelae. The pathogenesis is incompletely understood, hampering the development of an effective, adjunctive therapy, which is not available at present. Previously, erythropoietin (EPO) was reported to significantly improve the survival and outcome in a murine CM model. The study objectives were to assess myelin thickness and ultrastructural morphology in the corpus callosum in murine CM and to adress the effects of EPO treatment in this context.

**Methods:**

The study consisted of two groups of *Plasmodium berghei*-infected mice and two groups of uninfected controls that were either treated with EPO or placebo (n = 4 mice/group). In the terminal phase of murine CM the brains were removed and processed for electron microscopy. Myelin sheaths in the corpus callosum were analysed with transmission electron microscopy and stereology.

**Results:**

The infection caused clinical CM, which was counteracted by EPO. The total number of myelinated axons was identical in the four groups and mice with CM did not have reduced mean thickness of the myelin sheaths. Instead, CM mice had significantly increased numbers of abnormal myelin sheaths, whereas EPO-treated mice were indistinguishable from uninfected mice. Furthermore, mice with CM had frequent and severe axonal injury, pseudopodic endothelial cells, perivascular oedemas and intracerebral haemorrhages.

**Conclusions:**

EPO treatment reduced clinical signs of CM and reduced cerebral pathology. Murine CM does not reduce the general thickness of myelin sheaths in the corpus callosum.

## Background

In semi-immune individuals approximately 1% of all *Plasmodium falciparum* infections progress to cerebral malaria (CM); a cause of substantial morbidity and mortality worldwide [1]. Though the majority of surviving CM patients recover completely, a considerable fraction suffer from neurological sequelae. The sequelae are mostly transient and reversible, but 20-26% of children suffer from long-term cognitive impairment [2,3]. Neuronal injury with impaired axonal transport and cytoskeleton rearrangements are considered to contribute to sequelae [4]. In *post mortem* studies almost 40% of CM patients had spots of demyelination in the brain and brainstem [5,6], commonly associated with axonal injury [4]. The detection of myelin pallor and axonal injury has recently been linked to sequestration and ring haemorrhages in Malawian children [7]. Demyelination in human CM has been detected with histology and is considered to be secondary to axonal damage [4,6]. However, a recent study reports demyelination in areas without axonal injury but not vice versa and argues that the pathologies may be independent of each other [7].

For obvious reasons, demyelination in human CM can only be studied *post mortem* and may thus not be representative of the condition in survivors. Murine CM shares several similarities with human CM and offers the possibility to study the processes leading to demyelination and its functional importance [1]. However in murine CM, only limited studies on demyelination have been performed. One study has described demyelination in the optic nerve [8] and one reported apoptotic oligodendrocytes [9]. In the optic nerve, demyelination progressively worsened in the periphery of the optic nerve as clinical signs of CM appeared. In contrast, demyelination in the central part of the optic nerve was only detectable at a late stage of the infection. In addition, degenerated axons were apparent in terminally ill mice [8].

Several studies using the murine CM model have demonstrated behavioural changes prior to coma as CM progresses towards its terminal stage [10-14]. Thus, murine CM causes characteristic behavioural changes resembling cognitive impairment and loss of consciousness known from human CM [3,13,14]. Furthermore, mice surviving CM have long-term neurological impairment as human CM survivors do [3,13]. It is known from multiple sclerosis that severe demyelination and compromised remyelination cause cognitive impairment [15-17]. Strikingly, demyelination has received modest interest in CM. Thus, at present there is no evidence of a close link between cognitive impairment, axonal injury and demyelination, and the effects of adjunctive therapy in murine CM. Murine models are useful in addressing this aspect given that all pathophysiological aspects can be examined thoroughly and not only post mortem as is the case in human CM patients.

In this study, axonal myelination was assessed and quantified in the corpus callosum (CC) in murine CM by transmission electron microscopy. Moreover, axonal and vascular changes were assessed qualitatively. Furthermore, the effect of erythropoietin (EPO), which in previous murine studies provided neuroprotection [18-20], on changes in myelin and axonal structure was assessed.

## Methods

### Mice, parasites and infection

Pathogen-free, seven-weeks old, female C57BL/6 mice (Taconic, Ejby, Denmark) were kept under standard conditions with free access to standard pellet diet and water. *Plasmodium berghei* ANKA (PbA)-infected erythrocytes were stored in liquid nitrogen and used for the experiments after a single passage in acclimatized C57BL/6 mice. Mice were split in four groups with four mice in each (Table 1): uninfected, saline-treated: UninfSal; uninfected, EPO-treated: UninfEPO; infected, saline-treated: InfSal; and, infected, EPO-treated: InfEPO.

**Table 1:** Descriptive data of the grouping of mice and on the body temperature and clinical score at day 8 p.i

**Group**	**Injection at day 0 p.i.**	**Treatments day 4–7 p.i.**	**Body temperature day 8 p.i.**	**Clinical score day 8 p.i.**
UninfSal	Saline	Saline	37.9 ± 0.4	0 ± 0
UninfEPO	Saline	5000 IU EPO/kg	37.3 ± 0.9	0 ± 0
InfSal	10^4^ PbA	Saline	26.9 ± 1.1	3.25 ± 0.5
InfEPO	10^4^ PbA	5000 IU EPO/kg	37.2 ± .6	0.5 ± 0.6

All statistics are given as mean ± standard deviation (SD).

Briefly, two groups of mice (InfSal and InfEPO) were inoculated intraperitoneally (i.p.) with 10^4^ parasitized erythrocytes in 200 μl isotonic saline. UninfSal and UninfEPO received 200 μl isotonic saline only. Treatments were injected i.p. on day 4–7 post infection (p.i.) as 200 μl isotonic saline (control) or 5000 IU/kg recombinant human erythropoietin (EPO, Eprex®, Janssen-Cilag, Switzerland) diluted in 200 μl saline based on previously published results [19]. Body temperature was measured rectally (digital thermometer with rectal probe, Ellab, Denmark) and parasitaemia determined from thin Giemsa-stained blood smears by counting 500 erythrocytes.

The mice were observed daily for clinical signs of murine CM including convulsions, loss of balance and gripping reflex, paralysis, ruffled fur and lowered body temperature. A score was assigned from the clinical assessment (Table 2). On day 8 p.i. all InfSal mice showed clinical signs of CM and all mice were euthanized on this day.

**Table 2:** The clinical score of the mice was performed according to the table below

**Score**	**Observation**
0	No discernible clinical signs
1	Hunched back, slightly ruffled fur
2	Very ruffled fur, reduced rate of movement, developing motor impairments
3	Very ruffled fur, impaired balance/coordination, hypothermic, severe motor impairments such as ataxia, hemiplegia and paraplegia, convulsions, fitting
4	Very little movement, convulsions, fitting
5	Loss of consciousness/coma

### Tissue processing

A deep anaesthesia was induced by an i.p. injection of 10 μl/g body weight of Hypnorm®/Dormicum® [21] and the mice were then transcardially perfused with heparinized (15,000 U/l) isotonic saline for 30 sec. Tissue was perfusion-fixed with freshly made Karnovsky’s fixative (2.5% glutaraldehyde, 2% paraformaldehyde, 0.1 M Na-phosphate buffer, pH = 7.4) for 5 min at 30 ml/min. The brains were carefully split into two hemispheres through the midline and immersion-fixed for two hours in the same fixative before being transferred to 0.1 M Na-phosphate buffer and stored at 4°C. A pilot study was carried out to optimize fixative strength (glutaraldehyde content in Karnovsky: 3.5% vs. 2.5%) and immersion fixation time (two hours vs. 16 h).

### Transmission electron microscopy

One hemisphere was chosen at random, studied carefully and a part encompassing the whole cross-sectional area of the CC containing all nerve fibres was dissected and embedded in 5% agar. From the CC midline, 150 μm thick sections were cut on a vibratome perpendicularly to the long axis of the nerve fibres and stained with 1% toluidine blue to visualize nuclei and myelin sheaths. Using a dissection microscope the CC was carefully dissected from the surrounding tissue and split into parts not longer than 1 mm. Each part was dehydrated according to standard procedures, post-fixed in 1% Osmiumtetroxide (cat. no. 045384, Alfa Aesar, Germany) in 0.1 M Na-phosphate buffer (pH = 7.4) for 60 min, dehydrated in graded series of ethanol and embedded in epoxy resin (TAAB 812 Epon, TAAB, United Kingdom) using propyleneoxide as an intermedium. Ultra thin sections were cut on an ultramicrotome perpendicularly to the long axis of the nerve fibres in CC and collected on formware-coated 1x2 mm slot grids. The sections were stained with uranyl acetate (Ultrastain, Laurylab Saint-Fons Cedex, France) for 15 min, rinsed in distilled water and stained with lead citrate (Ampliqon, Skovlunde, Denmark) for 5 min and washed again. Pictures were taken at 7,900x magnification for stereology and at varying magnifications for qualitative assessments using a Philips CM10 electron microscope with a Morada digital camera connected to a workstation with SIS Analysis software (iTEM).

### Stereology

A 2-D fractionator design was applied to the digital images of the ultrathin sections, which contained all the nerve fibres of the CC cut perpendicular to their longest axis. The total number of axon profiles, *N(Axon),* is given as:

(1) $$N(Axon)=\frac{dx\cdot dy}{a(frame)}\cdot Q(Axon)$$

**Figure 1:**
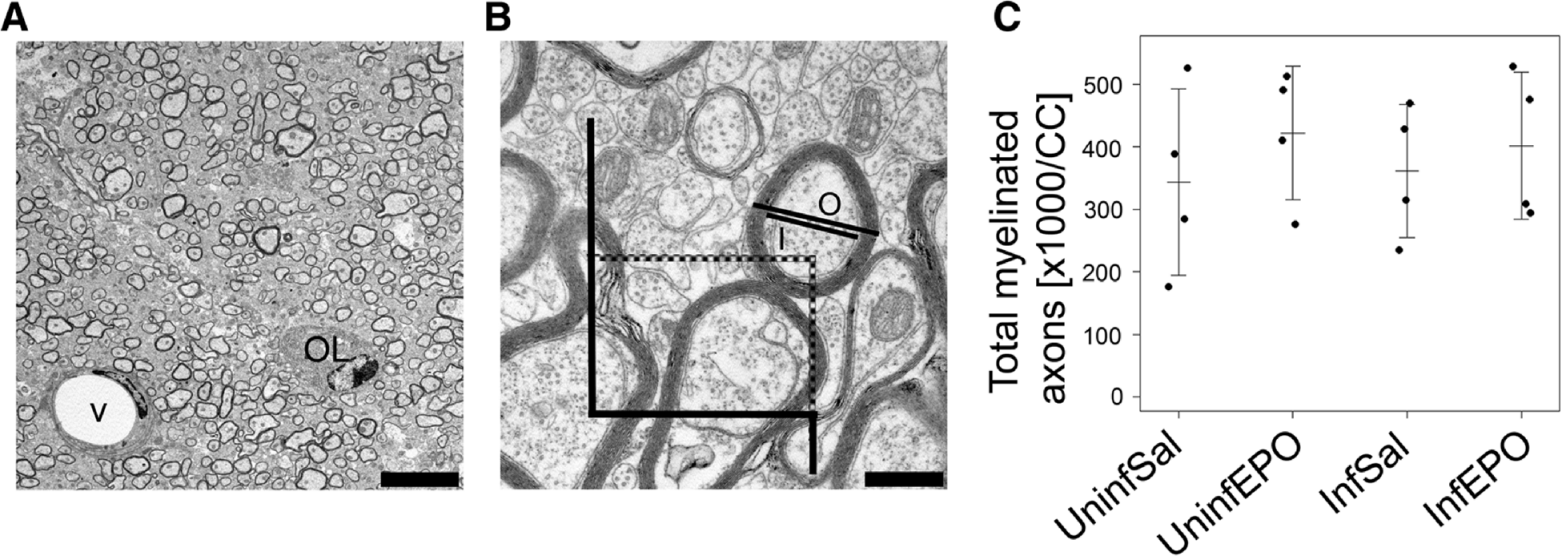
**Assessing myelination in the CC.** (**A**) Uninfected mouse. The lipid-rich myelin sheaths are electron-dense and myelinated axons appear as dark rings, also an oligodendrocyte (OL) and a vessel (V) are common findings. Scale bar corresponds to 5 μm. (**B**) At higher magnification, unmyelinated axons are seen interspersed between the larger myelinated axons. The counting frame is illustrated. Exclusion lines are in black, inclusion lines are dotted. Only one myelinated axon would be included in this frame; its inner (I) and outer (O) diameter was measured. Scale bar corresponds to 500 nm. (**C**) The total number of axons profiles at day 8 post infection was the same in all groups as depicted on the strip chart. Each dot represents the total number in one animal. Mean and SD are marked by a horizontal bar and whiskers, respectively.

*Q(Axon)* is the number of axon profiles counted in a 2D unbiased counting frame (Gundersen, 1977) with an area a(frame) equal to 16 μm^2^ or 32 μm^2^. The counting frames were positioned with a random start and a fixed distance between them in the x- (*dx*) and y-direction (*dy*) equal to 135 μm. Only axon profiles sampled by the counting frame were evaluated and at least 300 myelinated axon profiles were assessed in each CC. A practical example of how to measure the thickness of the myelin sheaths in transversely cut axons is shown (Figure 1). The myelinated axon profiles were counted and the fraction of profiles with myelin bulbs and other abnormalities were noted in addition to the numbers of axon profiles undergoing active myelination. The latter was identified by the dark cytoplasm of an oligodendrocyte encircling the axon profile completely. The thickness of the myelin sheaths was calculated from measuring the diameter of the axon with and without the myelin sheaths. Furthermore, the g-ratio was calculated (g-ratio = inner diameter/outer diameter). Since axons can appear elongated due to a slightly tilted section the axonal diameter and corresponding adjacent myelin sheath was always measured as the biggest diameter perpendicular to the longest axis of the axon profile. The microscopist was blinded for all investigations and assessments. By using design-based stereological sampling of axonal profiles, accurate results with an error variance of only 0.06 (Coefficient of error) when evaluating the noise effect were obtained.

### Qualitative assessments

The descriptive, histopathological assessments of the CC were performed after stereological analyses. They comprised detailed analyses of axonal myelination. Also, the axonal architecture was assessed and comprised studies of neurofilaments, microtubules and mitochondrial morphology. The endothelium was assessed for integrity of tight junctions, swollenness and intracellular organisation. Perivascular oedemas and haemorrhages were noted if present.

### Statistics

For continuous parameters, parametric ANOVA was used for group-wise comparisons and when appropriate, a Welch test with Holm correction was performed. Some parameters including axonal diameters were not Gaussian distributed and were normalized by log transformation: (x’ = log(x + 1)). Intergroup differences were considered significant if *p* < 0.05. All statistical analyses were performed using R (version 2.10.1 for Windows).

## Results

### Infection and treatment

**Figure 2:**
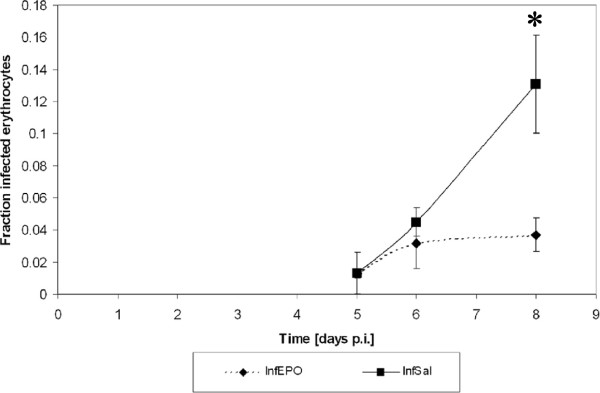
**Progression of parasitaemia.** Parasitaemia rose steadily in InfSal mice whereas it plateaued in InfEPO mice. At day 8 p.i., the parasitaemia was significantly higher in InfSal mice compared with infEPO mice (asterisk, *p* < 0.001). The dots denote the mean parasitaemia at the given day p.i.; the whiskers denote SD.

Parasitaemia increased progressively as previously shown [20]. EPO treated mice had a slower increase in peripheral parasitaemia (Figure 2) and at day 8 p.i the parasitaemia was significantly reduced in InfEPO mice (*p* < 0.001). At day 8 p.i., all InfSal mice were terminally ill and all animals were euthanized. In contrast no InfEPO mice showed signs of terminal CM. Terminally ill InfSal mice suffered from a sudden drop in body temperature, which was significantly lower than what was seen in the other groups (Table 1, *p* < 0.001). EPO-treatment improved this parameter significantly (*p* < 0.001) and resulted in a normal body temperature on day 8 p.i. There were significant differences in the clinical presentation between the groups (Table 1, *p* < 0.001). Notably, InfSal mice had poorer performance compared with InfEPO mice (*p* < 0.001). 

### Assessments of myelin sheaths

**Figure 3:**
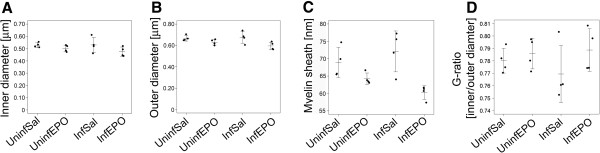
**Quantitative characteristics of the CC myelin sheaths.** The inner and outer diameter was measured as depicted in Figure 1B. (**A**) There was no significant difference in inner, i.e. axonal, diameter (*p* = 0.21). Each dot represents the mean diameter in one animal. Mean and SD is denoted by a horizontal line and whiskers, respectively. (**B**) There was a significant difference between the groups in outer diameter (*p* = 0.040). (**C**) In correspondence with this, there was also a significant difference in the myelin sheath thickness (*p* = 0.0039). (**D**) There was no change in g-ratio (inner diameter/outer diameter) between the groups (*p* = 0.39).

The number of myelinated axon profiles in the entire CC was assessed with transmission electron microscopy and the 2-D fractionator. The total number was similar in the four groups (Figure 1C, *p* = 0.79). The axonal diameter was measured and was similar in all four groups (Figure 3A, *p* = 0.74). The myelin thickness as well as the axon diameter plus myelin thickness (outer diameter) was different between the groups (Figure 3B, C; *p* = 0.0039 and 0 = 0.040, respectively), but the decrease noticed in the InfEPO group was not detected in post hoc tests (*p* > 0.074). There was no significant change in g-ratio between the four groups (Figure 3D, *p* = 0.39).

**Figure 4:**
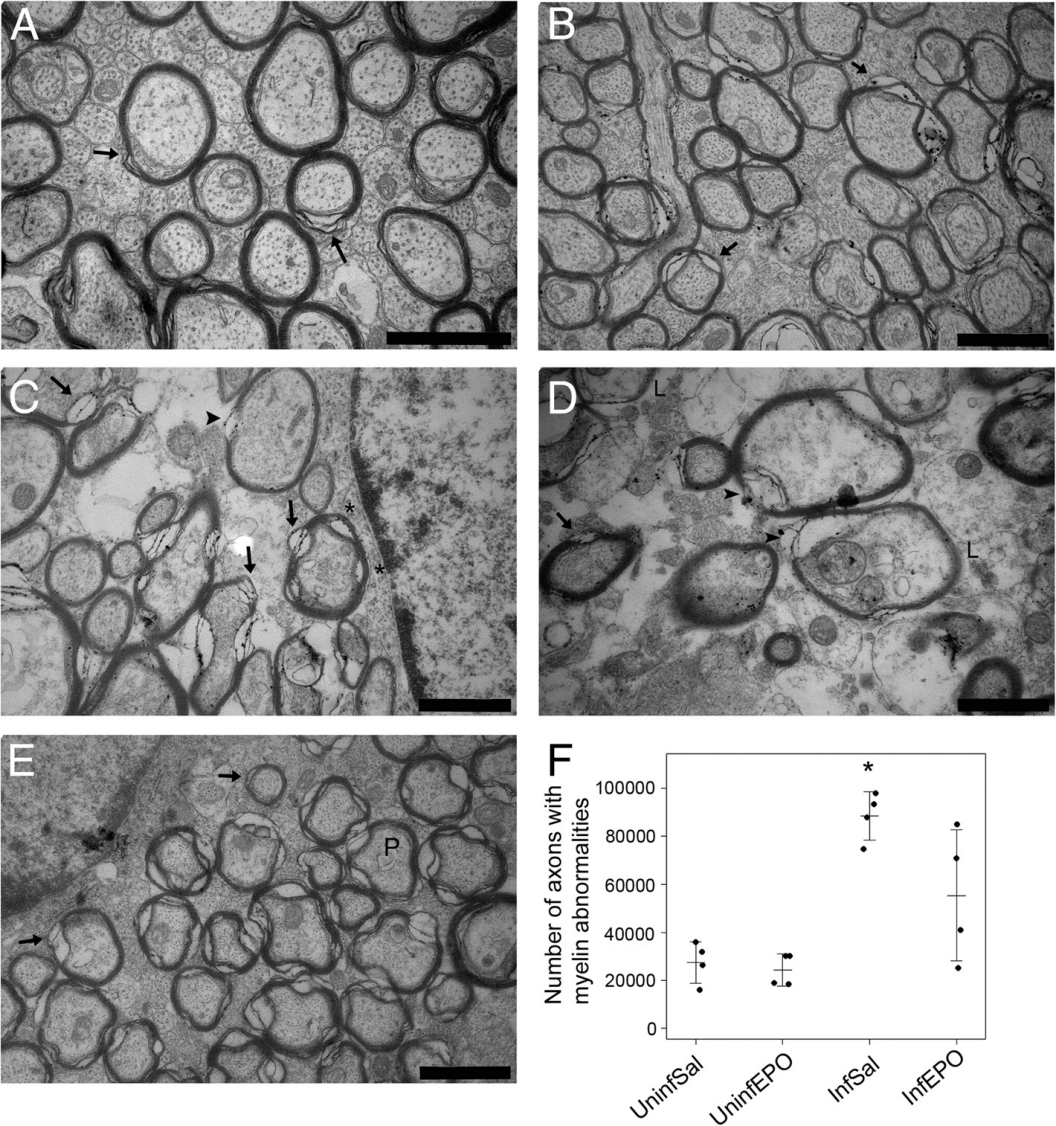
**CM induces pathological changes in myelin sheaths in the CC.** (**A**, **B**) The CC myelin sheaths in UninfSal (**A**) and UninfEPO (**B**) had normal appearance and compacted closely around the axon. In InfSal the myelin sheaths in the CC had several abnormalities (**C**, **D**). (**E**) In InfEPO the degree of abnormality was reduced. The most common abnormality was myelin splitting, which was also occasionally seen in uninfected mice (arrow in **A**, **B**, **C**, **D** and **E**). In infected mice, blebs of the myelin sheath (asterisks in **C**), complete, focal loss (arrowhead in **C** and **D**), and low electron density (L in D) were frequently seen. Increased periodicity was seen in InfSal and InfEPO mice (P in E). The denotations do not comprise all abnormalities present in each micrograph. Scale bars (**A-E**) correspond to 1 μm. (**F**) Quantification of the total number of abnormalities in the CC revealed a significantly higher number in InfSal mice as shown in the strip chart (*, *p* < 0.001). Each dot denotes the total number in one animal. The horizontal bars mark the mean values and the whiskers the SD.

The morphology of the myelin sheaths was assessed qualitatively (Figure 4). Normal myelin sheaths were wrapped tightly around the axons as seen in uninfected mice (Figure 4A and 4B). In InfSal mice a high number of abnormalities were seen (Figure 4C and 4D). Abnormal myelin sheaths comprised of six main morphological deviations: (1) blebs of the myelin sheaths, (2) splitting of myelin sheaths, (3) collapsed myelin sheaths (not shown), (4) a complete (and often patchy) loss of myelin, (5) increased periodicity of the individual sheaths, and (6) very low electron-density of the myelin sheaths. The latter abnormality gave a pale appearance representing a lack of unsaturated double bonds in the lipid-rich myelin sheaths. Splitting of the myelin sheaths was seen as increased periodicity in one focal spot of the transversely cut axon while increased periodicity was characterized by a general, increased distance between the individual sheaths wrapping the axon. The number of axons with abnormal myelin appearance was larger in InfSal mice than in other groups (Figure 4F, *p* < 0.001). Post hoc tests revealed that InfSal mice had significantly more axons with abnormal myelin appearance than uninfected mice disregarding treatment (*p* < 0.021). These events were not significantly more frequent than in InfEPO mice (*p* = 0.33); which were similar to uninfected mice (*p* > 0.27). The abnormalities were focally distributed and either one or several, neighbouring axons in a given area were affected. Axons in uninfected mice also had few axons without a completely tight myelin sheath but the abnormalities were restricted to myelin splitting.

**Figure 5:**
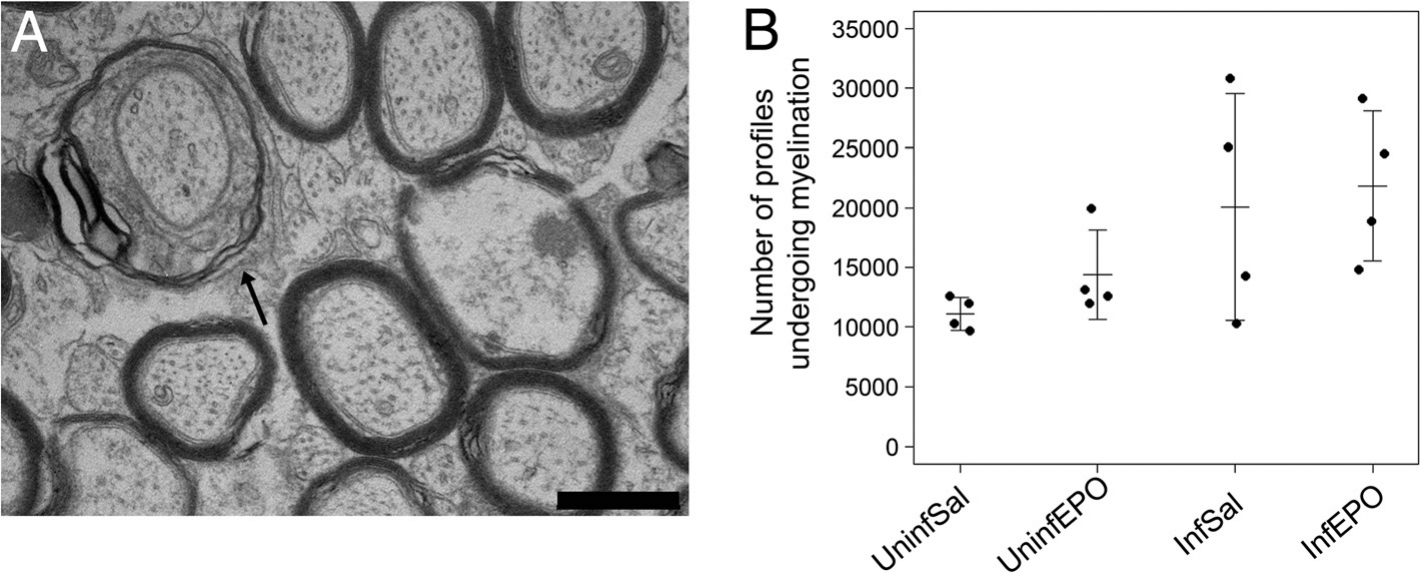
**Axon profiles being myelinated by oligodendrocytes.** (**A**) Oligodendrocytes encircling and myelinating axon profiles were frequently noticed in the CC (arrow). Scale bar corresponds to 250 nm. (**B**) The number of axonal profiles being myelinated in the CC was identical in all four groups (*p* = 0.063). Each dot denotes the total number in one animal. The horizontal bar marks mean values and the whiskers represent SD.

The initial phase of myelination, where an electron dense oligodendrocytic cytoplasm encircled an unmyelinated axon, was seen in all mice (Figure 5A) and no significant difference in the number of cell profiles where myelination took place was found between the groups (Figure 5B, *p* = 0.063).

### Axonal injury

**Figure 6:**
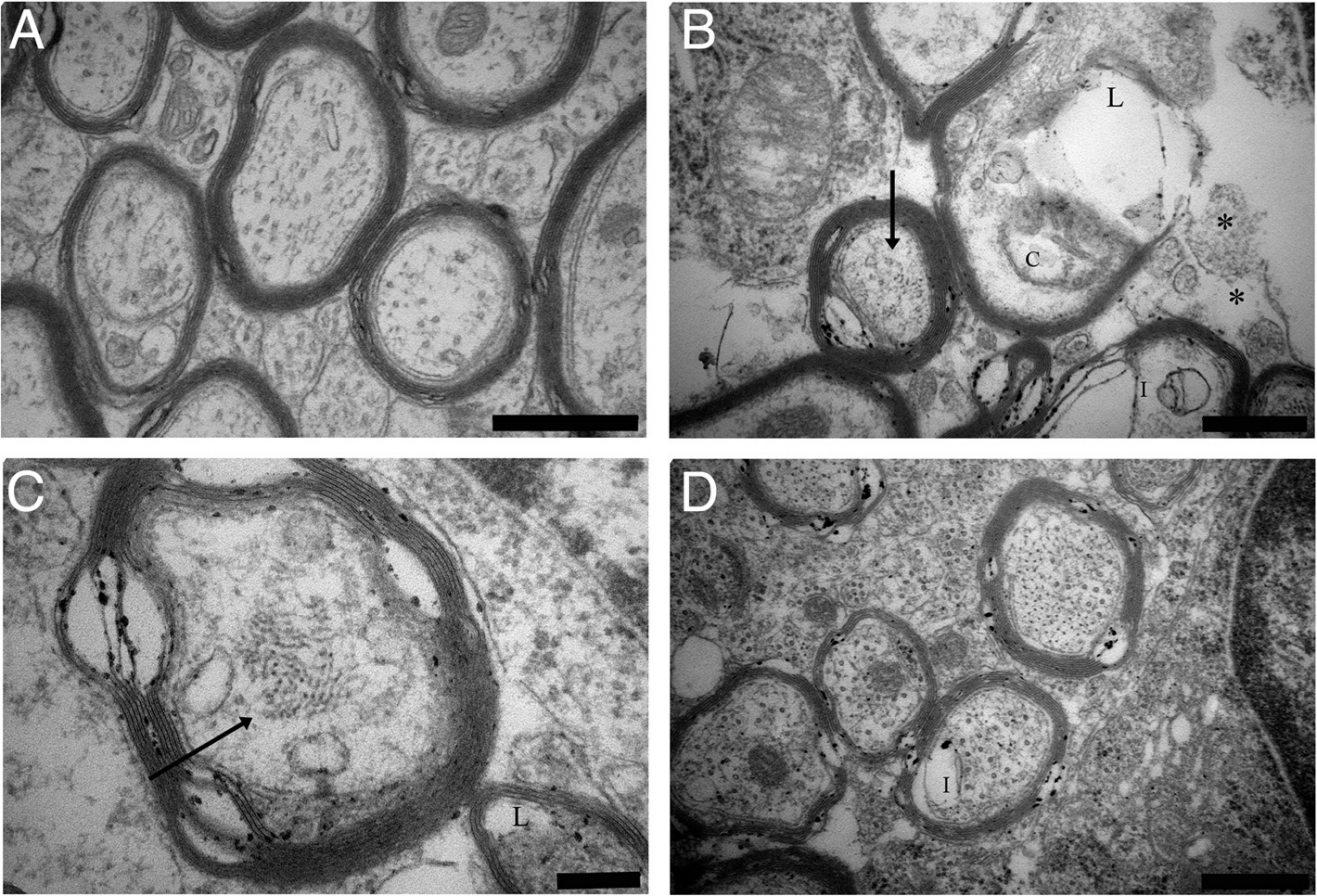
**Qualitative assessment of axonal injury in the CC.** Besides the changes in myelination, CM induced axonal injury. Uninfected mice regardless of treatment have healthy myelinated and unmyelinated axons in the CC (**A**). In the CC in terminally ill CM mice (**B**, **C**), several signs of axonal injury were detected including neurofilament aggregates (arrows in **B** and **C**), loss of axoplasm (L in **B**, **C**), inclusions in the axoplasm (I in B) and disprupted mitochondrial christae (**C** in **B**). Unmyelinated axons are heavily affected in terminal CM and if not lost, several unmyelinated axons have dysorganized cytoskeleton (asterisks in **B**). EPO treatment reduced axonal injury in the CC in both unmyelinated and myelinated axons (**D**). Inclusions of the axoplasm were seen (I in D). Scale bars correspond to 500 nm in **A**, **B** and **D** and 200 nm in **C**.

Axonal injury did not appear as prevalent as myelin sheath abnormalities. For the purpose of this study these changes were only described qualitatively. Generally, the cytoskeletal organisation was lost in InfSal mice. The specific type of injury detected was primarily seen as aggregates of neurofilament, inclusions in the axonal body and loss of axoplasm (Figure 6B and 6C); the latter likely due to proteolysis. More rarely, in some axons in InfSal mice, disrupted mitochondrial christae was noted (Figure 6B). The axonal injury was focal and most notably seen in InfSal mice. Most axonal injury was seen in axons with deranged myelin sheaths but in rare cases inclusions of the axonal body was seen in cells with normal myelin sheaths. Few axonal injuries were seen in InfEPO mice, consisting of inclusions of axoplasm (Figure 6D) and dysorganized neurofilaments (not shown). No axonal injury was observed in UninfSAL and UninfEPO (Figure 6A).

### Vascular changes

**Figure 7:**
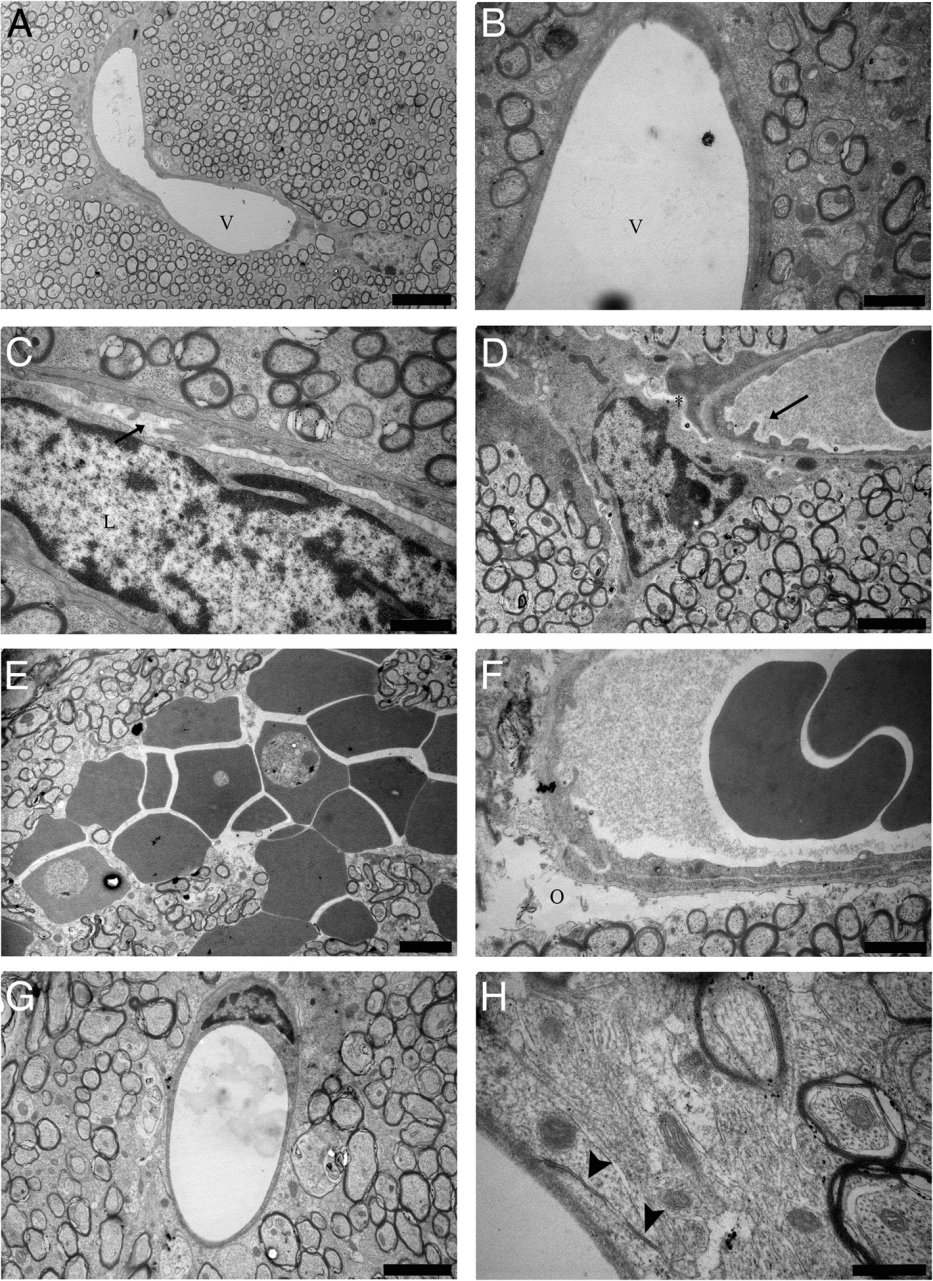
**CM induces changes to the endothelium and blood–brain barrier in the CC.** In uninfected mice regardless of treatment, the endothelium had a smooth appearance (**A** (UninfSal) and **B** (UninfEPO)). Vessel lumen denoted by V in **A** and **B**. In contrast, the endothelium of terminal ill CM mice had pseudopodic protrusions (arrows **C** and **D**) and often leukocyte adherence was noted (L in C). In some vessels the tight junctions were compromised and the astrocytic end feet did not support the endothelium (asterisk in **D**). Moreover, intracerebral haemorrhages into the CC were noted (**E**) and perivascular oedemas were frequent (O in **F**). In InfEPO mice, the endothelium had a normal appearance (**G**) and the tight junctions were intact (arrow heads in **H**). Scale bars correspond to 5 μm in **A**, 2 μm in **D**, **E** and **G**, to 1 μm in **B**, **C** and **F** and to 500 nm in **H**.

The endothelium in microvessels of the CC was similar in uninfected mice regardless of treatment. It had a smooth internal surface and was sealed with well-organized, tight junctions (Figure 7A and 7B). In InfSal mice the endothelium had several pseudopodic protrusions on its inner surface, and breakdown of tight junctions was noted (Figure 7C and 7D). These changes corresponded with the detection of perivascular oedemas, loosening of astrocytic end feet, adherence of leukocytes, plugging of vessels, and intracerebral haemorrhages (Figure 7E and 7F). The vascular compartment in InfEPO mice was well preserved and had a smooth internal surface comparable with uninfected mice (Figure 7G) as well as distinct and sealed tight junctions (Figure 7H).

## Discussion

The present study is the first study linking cerebral myelination and adjunct treatment in murine CM. Based on previous findings of CC infarcts [22,23] and motor-coordination impairments in murine CM [10,13] the CC was selected for ultrastructural assessment. As in human CM, mice with terminal CM had a significantly increased frequency of abnormal myelination. EPO treatment abrogated the pathophysiological events leading to this outcome and the myelination pattern was comparable with uninfected mice. This coincided with EPO treatment reducing clinical severity and peripheral parasitaemia, as previously shown [19,20]. The mechanism for reducing peripheral parasitaemia is currently being clarified but is likely the result of EPO’s effect on immune function [24] and its stimulating effect on the intraerythrocytic levels of nitric oxide [25].

The changes in axonal myelination were focally distributed in the CC and a reduced total number of myelinated axons or thinning of myelin sheaths in terminally ill CM mice was not seen. Defective myelin was characterized by focal blebs, splitting, collapsed sheaths and focal loss of fatty acids of the myelin sheath. The focal distribution of changes in myelination of the white matter is in line with the multifocal pathology of human and murine CM [4,6,8,9,21,26]. Splitting and blebs on the myelin sheaths were the most frequently observed abnormalities in infected mice and the former were occasionally seen in healthy uninfected mice. Intralaminar splitting of myelin sheaths has been reported in ischemia and may result from oligodendrocyte vulnerability to this insult [27,28]. Recently, widespread focal hypoxia in murine CM was demonstrated, which was counteracted by EPO treatment, suggesting a causative role of ischemia in the myelin damage observed in CM [29]. But the myelin damage may also result from oligodendroglial apoptosis, which is particularly frequent in the CC in this model [9]. Furthermore, demyelination can be caused by reactive oxygen species and pro-inflammatory cytokines, which are prevalent in CM [1,5,15,16,19,30,31]. EPO has been shown to reduce these processes in murine CM as well as in other neuropathologic conditions [17-19]. Unpublished, qualitative studies show deranged myelination in the cerebellum as well. This may contribute to impaired movement and ataxia in mice with CM. For quantitative studies the cerebellum is far from ideal due to the heterogenous and tree-like structures, which make it impossible to avoid detecting the same nerve fibre twice. These findings motivate future studies on linking neuro-motor impairments with the neuropathology observed.

The speed and degree of remyelination may be of importance for recovery from the neurological sequelae observed in human CM. In this study, it was noted that infected mice showed a tendency towards an increased number of axon profiles being myelinated but this requires further studies to be substantiated. The observed profiles being myelinated may either be normal age-associated myelination of unmyelinated axons [32] or remyelination after pathological demyelination. The increase observed in infected mice is likely due to remyelination induced by EPO and inflammation [17,33] and the lack of increase in some InfSal mice may be due to insufficient stimulation of remyelination pathways. EPO treatment stimulates oligodendrocyte progenitor cells and improves the functional outcome after experimental stroke [34] and experimental autoimmune encephalitis [17]. Thus, the current study calls for further characterizations of the effects of EPO on the occurrence and recovery from neurological sequelae after murine CM.

Abnormal myelination was observed more frequently than axonal damage. This suggests that demyelination is primary; i.e. takes place prior to axonal injury, but it may also indicate that oligodendrocytes are more susceptible than neurons to CM-induced apoptosis, ischemia and inflammation. However, the higher axonal vulnerability to hypoxia compared with glial cells [35] argues in favour of primary demyelination.

Axonal injury was most frequently seen as dysorganized or loss of the axoskeleton, inclusions of the axoplasm, and clearings of the axoplasm. Also focal periaxonal oedema was seen. These types of injuries have been described in previous studies of murine CM [8,9] and are hallmarks of cerebral ischemia. Axoskeletal changes are of importance since microtubules are essential for axonal transport and neurofilament maintain axonal integrity. Axoskeletal impairments can also be visualized by β-amyloid precursor protein (βAPP) accumulation [36] and indeed, βAPP accumulation has been shown in CM patients *post mortem*[6,7,37]. The axonal diameter was identical in the four groups in the study signifying that axonal swelling, resulting from cytotoxic oedema, was negligible.

EPO treatment protected axons from injury. In InfEPO mice, mitochondria, neurofilaments and microtubules appeared as in uninfected mice. Moreover, the smaller, unmyelinated axons were preserved and periaxonal oedemas were not present in EPO-treated mice. In murine CM, exogenous EPO reduces neuronal apoptosis and endogenous, cerebral production of EPO increases during the course of an infection highlighting a neuroprotective role for EPO in CM [18,19]. Neurons express the EPO receptor and EPO released from astrocytes can mediate cytoprotection in a paracrine fashion [38,39]. In human CM, the link between cerebral expression of stress markers and cognitive impairment has been proposed. Thus, the cognitive impairments particularly associated with CM, including attention and memory, could be closely associated with cerebral axonal injury and demyelination [2,3,40].

In terminal CM the vascular wall is affected and the blood–brain barrier (BBB) is compromised [7,22,41]. In CM mice, the changes in the endothelial compartment included pseudopodic protrusions, tight junction opening, perivascular oedemas and intracerebral haemorrhages. Pseudopodic endothelial cells may arise from low oxygen [42] and have been described previously in CM [8,9,41]. Interestingly, EPO-treated mice had a smooth endothelium, no apparent BBB impairment and no perivascular oedemas; comparable with that seen in uninfected mice. In a model of cerebral ischemia, EPO treatment reduced BBB leakage, increased the expression of tight junction proteins and reduced the formation of oedemas in the ischemic region [43]. A compromised BBB likely contributes significantly to CM pathogenesis and pathology, and BBB damage increases as CM progresses towards its terminal stage [44]. In the optic nerve it was noted that a high level of axonal injury and demyelination was seen in perivascular regions [8]; similar to human CM [6,45]. Other types of neuropathology show a link between BBB breakdown and abnormal myelination [28,46]. Likely, a combination of hampered perfusion in the cerebral microcirculation, BBB breakdown including oedemas and intracerebral haemorrhages as well as pro-inflammatory cytokines contribute to the neuropathology seen in this study.

Besides adding to the current understanding of CM pathology this study, to the authors’ knowledge, is the first to estimate the total number of myelinated axons in the murine CC. Previous studies have thoroughly evaluated the fraction of myelinated axons in the CC with regard to age [32]. Based on these observations, the seven-weeks old mice enrolled in this study would have approximately 13% of their CC axons myelinated [32]. The total number of axons in the CC appear to be an order of magnitude less than what is seen in cats [47]. The estimate given in this study is accurate and precise due to the sampling principles employed. Furthermore, the number of cells in the splenial part of CC is positively correlated with the size of the brain across species [48] and the estimate obtained alls within the expected range of myelinated axons in the CC.

## Conclusions

In human CM, abnormal myelination is present without axonal injury, pointing towards a more prominent role of demyelination than previously believed [7]. Moreover, a direct link between demyelination and sequelae has been suggested [49]. Acute CM probably does not result in demyelination and hampered remyelination comparable to that seen in chronic neuropathologies [4] but that does not preclude an important pathophysiological role of myelin abnormalities in CM. On the ultrastructural level, this study has documented frequent abnormal and defective myelination in the CC in murine CM. Also, axons and the endothelium are severely affected by murine CM. EPO treatment alleviated these CM-induced injuries.

The clinical relevance of EPO as adjunct therapy has not yet been sufficiently explored. Experimental data strongly suggests EPO to reduce pathology and severity [18-20,29] and its use as clinical therapy has recently been reviewed [50,51]. A clinical study in African children showed a negative correlation between plasma EPO levels and the risk of neurological sequelae [52] and a clinical safety trial using lower doses of EPO pr. kg bodyweight than employed in this study showed no adverse effects of the drug [53]. Thus, existing data collectively hint that EPO can reduce the acute CM-induced pathology as well as its sequelae, and may be used for adjunct CM therapy.

## Abbreviations

CC: Corpus callosum; CM: Cerebral malaria; EPO: Erythropoietin; InfEPO: Infected EPO-treated; InfSal: Infected saline-treated; i.p.: *Intra peritoneal*; PbA: *Plasmodium berghei ANKA*; P.i.: *Post infection*; UinfEPO: Uninfected EPO-treated; UinfSal: Uninfected saline-treated.

## Competing interests

The authors declare that they have no competing interests.

## Authors' contributions

CH performed animal experiments, designed the study, analysed the data, wrote the manuscript. PH participated in electron microscopic analyses and data analyses, edited the manuscript. TS designed the study and edited the manuscript. JRN designed the study and stereological analyses, analysed the data, edited the manuscript. JALK designed the study, analysed the data, edited the manuscript. All authors have read and approved the final manuscript.
